# Unconscious Weight Bias Among Nursing Students: A Descriptive Study

**DOI:** 10.3390/healthcare7030106

**Published:** 2019-09-12

**Authors:** Tracy P. George, Claire DeCristofaro, Pamela F. Murphy

**Affiliations:** 1 Department of Nursing 4822 E, Francis Marion University, Palmetto St. Florence, SC 29506, USA; 2 Behavioral Sciences Department 8620 Spectrum Center Blvd, Ashford University, San Diego, CA 92123, USA

**Keywords:** bias, nursing students, obesity, prejudice, activity, educational

## Abstract

There has been both an increase in obesity and anti-obesity bias in the United States. The Harvard Weight Implicit Association Test (IAT) is a reliable, valid test that can measure unconscious weight bias. First semester Bachelor of Science in Nursing (BSN) students were surveyed anonymously mid-semester and at the end of the semester after completing the Harvard Weight IAT. Sixty-nine out of 77 students completed pre- and post-surveys. Weight preference towards others was not shown to be related to the respondent’s own self-reported body mass index (BMI). The majority of respondents exhibited more weight-related bias on the IAT than they realized. The three qualitative themes that emerged included Awareness of Personal Beliefs and Stereotypes, Reminder to be Impartial, and Skepticism about the IAT. It is important for undergraduate nursing students to be aware of possible unconscious weight bias in order to provide high-quality care to patients.

## 1. Introduction

Unconscious bias may contribute to the health disparities that exist in the United States [1]. Currently over 60% of Americans are categorized obese [2], and this is relevant due to the increase in anti-obesity bias in the United States [3]. Obese patients may be hesitant to access health care services due to concerns about provider weight bias [4].

There is increasing research on obesity bias in healthcare professionals and students in healthcare disciplines. In a study of 66,799 volunteers taking the Thin-Fat Implicit Association Test (IAT) on the Harvard Project Implicit^®^ website, weight-related attitudes and beliefs were significantly correlated with implicit anti-fat bias [5]. Studies indicate that this bias may extend to physicians and medical students [6,7]. Other health professionals may hold weight bias, including registered dieticians, diagnostic radiographers, and pharmacists [8,9,10].

Nurses and nursing students may also hold unconscious biases against obese patients. When asked about attitudes of prejudice, a qualitative theme of Prejudice against Obese Individuals emerged in a study involving 50 undergraduate nursing students [11]. In a study of 45 undergraduate nursing and 45 undergraduate psychology students, both groups of students exhibited implicit bias toward overweight using the IAT [12]. Ward-Smith and Peterson found some nurse practitioners have negative attitudes about overweight and obese patients [13].

It is important to consider both the implicit and explicit biases of students in health care programs. In a study of 354 third-year medical students using the IAT, 33% reported that they had moderate-to-strong explicit anti-fat bias. In the same study, 39% had an implicit anti-fat bias, and 67% of students did not realize that they held an implicit anti- fat bias [14]. Additionally, in a longitudinal study looking at 1795 first- and fourth-year medical students, implicit weight bias decreased, but explicit weight bias increased a small but statistically significant amount [15]. The increase in explicit weight bias may indicate a decline in empathy toward obese patients during medical education.

Encouraging students to become more aware of their unconscious bias is important and becoming aware of such bias can be accomplished with implicit bias testing [16]. In healthcare professional education, some suggested strategies to combat bias against obese patients include positive experiences with obese patients, elimination of discriminatory behavior by faculty and residents, and curricular changes [15]. Suggested provider strategies involve recognition of the complexity of conditions associated with obesity, communicating to staff and patients the awareness that obesity is not a character defect nor personal failing, educating patients that small weight losses can lead to large health benefits, and consciously creating an environment that is comfortable for overweight and obese patients (armless chairs, appropriate equipment and gowns) [4].

IATs measure unconscious beliefs that people hold [17]. This type of test has been used frequently in a variety of fields, but it has been utilized less often in nursing research. The IATs are reliable, valid tests that may provide useful data in nursing research [17]. One author’s experience teaching graduate nursing students in a doctoral program resulted in our interest in this topic. In a fully online asynchronous pharmacology course, a required discussion board asked students to complete the Harvard Weight IAT and then compare their predicted result with the actual IAT results. The majority of students who were found to have a weight bias (thin preference) based on their IAT results had predicted that they were neutral in regard to weight and were dismayed to find that they did have an unconscious bias against obese individuals. None of these students, all practicing nurses, admitted to any knowledge of biased delivery of care in the clinical setting but wondered if perhaps they were unaware of such behaviors. This reflection resulted in a rich discussion regarding the value of knowing if one had an implicit bias, and many students went on to complete additional tests on the Project Implicit^®^ website. However, a significant minority of students became angry, using strong verbiage to denounce the validity of the IAT and the value of the learning activity. These were students who had predicted they were neutral, but in fact had an IAT score that demonstrated a thin preference. No amount of explanation by the instructor, nor by classmates, of the validated nature of the IAT or the value of being aware of bias had an impact on these students. Based on this experience, the instructor recognized that such a learning activity would be of value at an earlier level of nursing education and could be considered an aspect of cultural sensitivity training.

The purpose of this study was to determine whether nursing students’ explicit beliefs about weight preference were consistent with the implicit attitudes from the IAT, as well as to obtain student perceptions about the IAT. A secondary focus was to determine if weight bias is associated with the individual’s own weight category, as determined by body mass index (BMI) calculated from self-reported height and weight.

## 2. Materials and Methods

The study setting is a public liberal arts university in the southeast United States. The study was approved by the university institutional review board (IRB) (IRB Number George-04-12-2016-003). The students in the study were first semester junior Bachelor of Science in nursing (BSN) students in a health assessment course. Demographic information and survey data were obtained via anonymous surveys to assess their predicted weight bias, as well as their cultural competence and communication prior to and after completing the Harvard Weight IAT [18]. The Harvard Weight IAT is a freely available validated test that measures the strength of associations between obese and thin people. The scoring is online and provides a person’s unconscious preference for obese and thin people. No names were on the surveys, but students used a numerical identifier so that the pre- and post-survey data could be compared. The survey was adapted with permission from an existing 12-question survey that was utilized to assess cultural competence and communication before and after pharmacy students completed a cultural competence course [19]. For this study, Assemi and colleagues’ survey was reduced to eight questions to better reflect nursing care [19]. In Assemi and colleagues’ study, the Cronbach alpha was 0.84 for the pre-survey, while it was 0.87 for the post-survey; however, statistical analysis was not performed after it was modified for use with nursing students.

Mid-semester, the department administrative assistant obtained consent and administered the pre-survey. At the end of the semester, the Harvard Weight IAT was completed in class by all students as part of routine class work. After completing the Harvard Weight IAT, students completed the post-survey. Participation in completing the pre- and post-surveys was voluntary. Students provided their IAT result as part of the post-survey. In addition, students self-reported their own height and weight, from which their individual BMI was calculated. Descriptive statistics were calculated for demographic factors of participants. The Wilcoxon signed rank test was used to compare respondents’ ratings of their weight preference/bias to their scores on the Harvard Weight IAT. Spearman correlations were performed to determine whether there were significant bivariate relationships among respondent’s BMI, self-reported weight preference/bias, IAT result, and whether or not the respondent was surprised by the IAT result. In addition, qualitative questions were included on the post-survey to determine the students’ perceptions of the IAT. The three qualitative questions in the post-survey included the following: (1) Did you find your IAT result to be surprising? If so, how?; (2) How do you think taking this test and/or its results will influence your professional dealings with patients in the clinical setting?; and (3) Please provide your comments on the IAT.

## 3. Results

### 3.1. Demographics

Sixty-nine out of 77 students in the health assessment course took both the pre- survey and the post- survey, for a response rate of 89.61%. Self-report data indicated that 10 students were male and 59 were female, and the majority of respondents were Caucasian *(n* = 56), followed by African American (*n* = 10), Asian *(n* = 1), and Other (*n* = 2).

### 3.2. BMI

The individual respondent BMI was calculated based on self- reported height and weight, and ranged from 18.14 to 38.41, with a mean of 24.77 and a standard deviation of 4.3118. A BMI Range variable was created to code the BMI into four categories: underweight (BMI < 18.5, *n* = 1), normal (BMI ≥ 18.5 < 25, *n* = 40), overweight (BMI ≥ 25 < 30, *n* = 19), and obese (BMI ≥ 30, *n* = 9).

### 3.3. Prediction of Weight Bias

In terms of students being able to predict their weight preference/bias, pre-survey results were compared to the actual self-reported IAT results given on the post-survey. Regarding the presence of weight bias, a Wilcoxon signed rank test found a significant difference between the pre-survey statement about preferences for fat or thin people (student’s individual prediction of bias status) and their actual IAT result (Z = −4.629, *p* < 001). See Table 1 for a summary of data.

### 3.4. Those Predicting Neutral/No Weight Bias

Fifty-six out of 69 students (81.16%) predicted they had no weight bias, but this self-assessment prediction was confirmed by the actual IAT results for only 11 (19.64%) out of the 56 respondents. Of the 45 respondents (65.22%) who incorrectly predicted their lack of weight bias, five had some degree of preference for fat people over thin people, and 40 preferred thin people over fat people to some degree. Specifically, for these 45 students, their actual IAT results showed one strongly preferring fat people, three moderately preferring fat people, one slightly preferring fat people, 15 slightly preferring thin people, 11 moderately preferring thin people, and 14 strongly preferring thin people.

### 3.5. Those Predicting Personal Weight Bias

Twenty-one out of 69 students (30.43%) correctly predicted the results of their Weight IAT (11 students correctly predicted neutral/no preference, nine correctly predicted a preference for thin people, and one correctly predicted a preference for fat people). In addition, of those students predicting they had a weight bias, three students incorrectly predicted their type of bias (one thought they preferred fat people but actually preferred thin, and two thought they preferred thin but they were actually neutral).

Of interest, weight preference in others was not shown to be related to the respondent’s self-reported weight status (BMI). However, a significant Spearman correlation indicated that participants’ whose IAT score showed a preference for thin people were more likely to be surprised by their score (ρ = 295, *p* = 014). In summary, the majority of respondents exhibited more weight-related bias on the IAT than they predicted, thus demonstrating unconscious weight bias.

### 3.6. Qualitative Themes

The qualitative responses were analyzed by two authors using directed content analysis, and any differences were discussed. There were 68 responses to question 14, 66 responses to question 15, and 45 responses to question 16. The three qualitative themes that emerged included Awareness of Personal Beliefs and Stereotypes, Reminder to be Impartial, and Skepticism about the IAT. For the theme of Awareness of Personal Beliefs and Stereotypes, one student stated, “It was an eye opener on how I really feel about fat versus thin.” Another student said, “You could change your views and be able to better perform care.” Statements in this theme focused on the student’s recognition of potential bias, despite self-assessment to the contrary.

Regarding the theme of Reminder to be Impartial, one student said, “It can help me recognize bias and help everyone equally.” Another nursing student stated, “It makes me more aware of my feelings toward certain people. My nursing care should be equal towards everyone.” The emphasis of statements within this theme was the importance of impartiality towards patients in the delivery of care.

Several students wrote comments that indicated skepticism about the actual Weight IAT itself. This skepticism took the form of dismissing the IAT results or being unsure of the accuracy/reliability of the IAT instrument. For instance, some students felt that the IAT results would have no influence on their personal interactions with patients because they were already treating all patients equally. A student reported, “It will not influence me because I see people the same.” One student stated about the IAT, “I feel it does not accurately reflect feelings.” Another student said, “I don’t see how the pictures and words correlate to preferences to people and their appearances.”

## 4. Discussion

Among nursing students, there is a need for heightened awareness of unconscious bias against obese individuals so that culturally appropriate care can be provided to patients. In this study, over 65% of nursing students incorrectly felt that they had no bias against fat people, and there was a significant difference between the pre-survey statement about their predicted neutrality towards weight (preference for fat or thin people) and the student’s actual IAT result. This is similar to the results of Miller and colleagues, where the majority of medical students did not realize that they held an implicit anti-fat bias [14].

Implicit weight bias involves rapid and automatic responses to obesity and affects both the well-being of oneself as well as others. Weight bias often includes negative assumptions about the personal characteristics of those with obesity, reinforcing stereotyping and weight stigma [20]. Of interest to healthcare professionals, it has been found that obesity stigma can drive health disparities, with those experiencing weight-related discrimination more likely to become and stay obese in the long term [21]. Additionally, patients who are obese may be less likely to seek medical care because of their concerns about obesity bias [4]. Thus, weight-related bias is important to recognize at the student level in order to help reduce bias-associated behaviors.

With the availability of psychometric tools to assess implicit attitudes, educators can provide learning activities to objectively measure unconscious bias. These learning activities can include offering students access to bias testing and results, creating opportunities to discuss predicted bias and actual bias findings, and encouraging self-reflection on explicit and implicit biases. Curricular content can include facilitation of types of common biases, how bias originates, and ways to adjust behaviors to account for recognized bias. Various forms of activities can be employed, with a goal of reducing the potentially negative impact of unconscious bias on quality of patient care, interprofessional relationships, and clinical professionalism.

These learning activities can include seminar discussions, assigned readings, and simulation training with scenarios. Incorporating these activities into existing related content (e.g., cultural diversity, sexual orientation) will emphasize the mainstream aspect of bias awareness. Some learning institutions have implemented modules based on evidence-based psychotherapy, such as Acceptance and Commitment Therapy (ACT). It is important to recognize that such bias testing may result in categorizing students as being “pro” or “anti” a group. This may reduce student acceptance of the IAT results and may also cause considerable distress. Implications for educators, therefore, include the caveat to avoid forcing students into such categories [22]. Authors should discuss the results and how they can be interpreted in perspective of previous studies and of the working hypotheses. The findings and their implications should be discussed in the broadest context possible. Future research directions may also be highlighted.

## 5. Conclusions

What we have learned from this study is that although nursing students may believe themselves to be free of weight bias, implicit weight bias does exist in a large percentage of the student body. Recognizing and addressing this bias through carefully crafted learning activities can help reduce unconscious prejudicial behaviors, thus improving quality of care. Implications for educators include the recommendation to administer validated tests for implicit weight bias and facilitate understanding of the results in the context of diversity and cultural sensitivity activities in order to promote quality care. Limitations to this study include a single group of undergraduate nursing students from one university. Additional research needs to be done on weight bias among nursing students at the undergraduate and graduate levels.

## Figures and Tables

**Table 1:** Predicted vs. actual weight bias. IAT = Implicit Association Test.

Pre-Survey	IAT Prefer Fat	IAT No Preference	IAT Prefer Thin	Totals
Prefer Fat	1	0	1	2
No Preference	5	11	40	56
Prefer Thin	0	2	9	11
Totals	6	13	50	69
